# Hierarchical Assessment of Mutation Properties in *Daphnia magna*

**DOI:** 10.1534/g3.118.200472

**Published:** 2018-08-29

**Authors:** Sarah Eberle, Djeneba Dezoumbe, Rhegan McGregor, Shane Kinzer, Whitney Raver, Sarah Schaack, Leigh C. Latta

**Affiliations:** * Lewis-Clark State College; Division of Natural Sciences and Mathematics; Lewiston, ID 83501; † Reed College; Department of Biology; Portland, OR 97202

**Keywords:** mutation accumulation, mutational bias, behavior, evolvability, genetic drift

## Abstract

Understanding the context-dependence of spontaneous mutations is crucial to predicting evolutionary trajectories. In this experiment, the impact of genetic background and trait-type on mutational susceptibility was investigated. Mutant and non-mutant lines of six unique genotypes from two populations of *Daphnia magna* were phenotypically assayed using a common-garden experiment. Morphological, life-history, and behavioral traits were measured and estimates of the mutation parameters were generated. The mutation parameters varied between the populations and among genotypes, suggesting differential susceptibility to mutation depending upon genomic background. Traits also varied in their susceptibility to mutation with behavioral traits evolving more rapidly than life-history and morphological traits. These results may reflect the unique selection histories of these populations.

Spontaneous mutations, uncorrected errors that occur during DNA replication and repair, are the ultimate source of genetic variation. Small fractions of incoming mutations are beneficial, and provide the fuel for adaptive evolution (Bataillon 2000; Eyre-Walker and Keightley 2007; Perfeito *et al.* 2007). However, a majority of spontaneous mutations are either neutral or deleterious with respect to fitness (Eyre-Walker and Keightley 2007; Halligan and Keightley 2009). Normally, purifying selection eliminates a majority of the deleterious variants from a population, leaving behind the beneficial and neutral mutations. However, in populations where selection is relaxed or eliminated genetic drift governs the fate of incoming mutations. Unlike selection, which fixes beneficial mutations in a population, genetic drift results in the stochastic fixation of mutations, regardless of the effect of those mutations (erratum: Nature 453: 128) on fitness. Given that the deleterious mutation rate is much higher than the beneficial mutation rate (Kibota and Lynch 1996; Eyre-Walker and Keightley 1999; Denver *et al.* 2004; Haag-Liautard *et al.* 2007), populations that evolve under genetic drift accumulate deleterious mutations, and experience a gradual erosion of fitness. In populations where conditions of relaxed selection persist for extended periods, the interaction between genetic drift and deleterious mutations can ultimately result in the extinction of the population through mutational meltdown (Lynch *et al.* 1993; Lande 1994; Lynch *et al.* 1995; Zeyl *et al.* 2001).

Despite the importance of mutation in shaping the attributes of natural populations, our understanding of the context-dependence of mutation parameters, particularly the phenotypic effects of mutations, is still limited. Current evidence indicates that mutational effects vary in different environments. For example, mutational effects can be exacerbated under stressful environments (*e.g.*, Kondrashov and Houle 1994; Latta *et al.* 2015), particularly when the environmental stressor is high population density (Agrawal and Whitlock 2010). However, non-density forms of environmental stress have varying influence on mutational effects, including reducing and even eliminating the effects of mutation on fitness (Agrawal and Whitlock 2010). Additionally, there appears to be genotypic variation in the spontaneous mutation parameters. For example, some genotypes within a species have high mutation rates, while other genotypes have low mutation rates (Demerec 1937; Haag-Liautard *et al.* 2007; Latta *et al.* 2013). Finally, traits that differ in their genetic architecture are differentially susceptible to the effects of mutation accumulation (MA) (Houle 1992; Houle *et al.* 1996; Rowe and Houle 1996; Lynch *et al.* 1999; Halligan and Keightley 2009; Latta *et al.* 2015). Specifically, traits with complex genetic architectures controlled by numerous genetic loci, such as life history traits, appear to be more prone to MA than traits with simple genetic architectures, such as morphological traits (Houle 1992; Latta *et al.* 2015). Additionally, evidence suggests that quantitative traits have higher mutational heritability than gene expression traits, which may be due to the large number of genes controlling quantitative traits, or because gene expression traits are more sensitive to environmental variation (Rifkin *et al.* 2005; Huang *et al.* 2016).

Genotypic variation in the mutation parameters has been demonstrated in several organisms including *Drosophila melanogaster* (Haag-Liautard *et al.* 2007), *Chlamydomonas reinhardtii* (Kraemer *et al.* 2017), *Daphnia pulicaria* (Latta *et al.* 2013), and various species of rhabditid nematodes (Baer *et al.* 2005). The patterns that have emerged from these studies suggest that differences in the underlying mutation rates among genotypes, and/or differences in the epistatic interactions between new mutations and the genomic background in which they arise, may contribute to variation in estimates of mutational effects (Remold and Lenski 2004; Weinreich *et al.* 2005; Phillips 2008; Le Gac and Doebeli 2010; Ness *et al.* 2015; Kronholm *et al.* 2017). One genomic feature that predicts genotypic variability in the mutation parameters is the pre-existing load of deleterious mutations the genotype harbors. Several studies report increases in mutation rate estimates in genotypes with high initial mutation loads (Ávila *et al.* 2006; Agrawal and Wang 2008; Sharp and Agrawal 2012), which may contribute to rapid changes in fitness in these loaded genotypes.

Trait variation in mutation rates and effects has also been demonstrated in numerous systems. The working hypothesis is that physiologically complex traits controlled by numerous genetic loci present large mutational targets because the number of genetic loci controlling a trait is directly proportional to the mutation rate for that trait (Houle 1992; Houle *et al.* 1996; Rowe and Houle 1996; Latta *et al.* 2015). For example, fitness traits display greater mutational susceptibility than morphological traits, putatively due to the complex genetic architecture of life-history traits relative to morphological traits. One set of traits that may be especially susceptible to mutation due to its complex genetic architecture are those under the control of the nervous system, such as behavioral traits. The effect of spontaneous mutation on neural function, and on behavior specifically, has largely been neglected with most phenotypic studies focusing on life-history and morphological traits (*e.g.*, Azevedo *et al.* 2002, Estes *et al.* 2004; Latta *et al.* 2015). However, the effect of mutation on behavioral traits has been investigated in a few systems. In *Caenorhabditis elegans*, behavioral performance declines at a rate similar to fitness traits (Ajie *et al.* 2005; Estes *et al.* 2005). Similarly, MA lines of *Drosophila* are less motile than associated control populations (Latimer *et al.* 2014), and mutations have a stronger effect on male reproductive performance than female reproductive performance (Mallet and Chippindale 2011; Mallet *et al.* 2012; Sharp and Agrawal 2013; Almbro and Simmons 2014), both of which suggest these observations may be explained by differences in the mutational target size of the behavioral traits investigated.

To examine genotypic and trait-specific variation in susceptibility to the effects of mutation, we conducted an MA experiment on six unique genotypes of *Daphnia magna* isolated from a broad latitudinal gradient. *Daphnia* are an ideal system for MA experiments as they can be maintained clonally in the lab, which allows the accumulation of mutations in a heterozygous state in naturally occurring genomes (Keith *et al.* 2016). Following MA, a phenotypic assay in which life-history, morphological, and behavioral traits were measured was used to compare the performance of the mutation lines and controls. This experimental design allows us to use a hierarchical approach to simultaneously examine both genotypic and trait-specific variability in mutational effects, and test the nature of the context-dependence of spontaneous mutation.

## Materials And Methods

### Study System

The aquatic microcrustacean *Daphnia* is a model organism for ecological and evolutionary biology studies (*e.g.*, Miner *et al.* 2012). The cyclical parthenogenetic nature of *Daphnia* makes them an ideal organism to use in MA experiments because clonal reproduction can be maintained in the lab using specific environmental conditions. In these experiments, *D. magna* were reared under a 16L:8D photoperiod at a constant temperature of 18°.

The *D. magna* genotypes used in this experiment were collected along a latitudinal gradient that captures a range of environmental variation including temperature and photoperiod (Table S1). Three unique genotypes from each of two populations (Germany and Israel) were used to initiate the control and mutant lines. These populations capture variation in local selective pressures that may influence the phenotypic effects of mutation. Importantly, this sampling design permits an assessment of mutation parameter variability both among genotypes within a population, and among populations.

The stock cultures for each genotype were maintained in 250 mL beakers containing 175-200 mL of Aechener Daphnien Medium (ADaM; Klüttgen *et al.* 1994) under a constant photoperiod (16L:8D) and temperature (18°), and fed the unicellular green alga *Scenedesmus obliquus ad libitum* (2-3 times per week). One concern in MA experiments is the maintenance of a stable control during the MA phase of the experiment. In *Daphnia*, control lines are maintained by establishing populations of large size, which minimizes the number of incoming deleterious mutations (Flynn *et al.* 2016). However, this approach also generates opportunity for control lines to adapt to the lab environment during the MA phase. In order to minimize the possibility of adaptation in the control populations during the MA phase we maintained stock cultures of each genotype for 6 months under these lab conditions prior to initiation of the MA experiment.

### Mutation Accumulation Experiment

Control and mutant lines were initiated from clonally produced offspring of a single asexual female isolated from the stock cultures of each genotype (Table S1). Control lines were maintained in two replicate 3 L jars containing 2 L of ADaM, under constant temperature (18°) and photoperiod (16L:8D), and fed the unicellular green alga *S. obliquus ad libitum*. The media in the jars was replaced every 2-3 weeks, and individuals in the two replicate jars were mixed to maintain as much genetic homogeneity among the jars as possible. The maintenance of control lines in large jars ensures that population densities, which varied between several hundred to a few thousand individuals, were high enough that new mutations with deleterious effects should be efficiently eliminated from the populations by purifying selection (Flynn *et al.* 2016).

Mutant lines for each genotype were established by generating five replicate clonal lineages from each of six genotypes (30 lineages total). These lineages (MA lines) were initiated by placing a single clonally produced female in a 250 mL beaker containing 100 mL of ADaM supplemented with *S. obliquus* at a concentration of 600,000 cells/mL. All MA lines were maintained in conditions identical to the control lines (16L:8D, 18°). The food/media mixture in each beaker was replaced once per week, and each line was fed a prescribed volume of concentrated *S. obliquus* three days after the media replacement to reset the algal cell concentration in the beaker to 600,000 cells/mL.

Each MA line was propagated from generation to generation over the course of the experiment via single offspring descent by taking a single juvenile from the second clutch of the mother. A series of backups were maintained in parallel with the focal lineages in the event that the single individual intended to be used to establish the next generation died before reproduction, or was a male. In the event that the focal lineage and all backups either died before reproduction, or were all males, the lineage was declared extinct and a new replicate lineage was established from the control lines. In this experiment there were two mutant lines, both descended from a single German genotype, which were restarted from the control population. For these lines, the number of generations of divergence from the control population was calculated from the time of line re-initiation. Thus, our estimates of the number of generations of divergence presented here represent the number of generations in which lines evolved under genetic drift after isolation from the control population. The two lines that were restarted from the control populations undoubtedly experienced mutation accumulation while in the control populations. However, individuals in control populations are under strong purifying selection (Flynn *et al.* 2016), so the mutations that these lines accrued while in the control population should be predominantly neutral with respect to fitness. In total, the MA phase of the experiment was conducted for approximately 2.5 years and resulted in an experiment-wide average of 22 generations of divergence between MA lines (Table S2).

### Phenotypic Assay

Life-history, morphological, and behavioral traits for mutant and control individuals were assayed simultaneously using a common-garden experiment. Single juvenile females (five for each MA line, and 15 for each control line) were isolated from the MA lines or control populations and placed in 150 mL beakers containing 100 mL of ADaM supplemented with *S. obliquus* to yield a concentration of 600,000 cells/mL. Beakers were randomized on trays and placed in an environmental chamber under standard laboratory conditions (16L:8D, 18°). Over the course of the assay the food/media mixture was replaced every other day to ensure individuals had sufficient food, and the trays containing beakers were rotated in the environmental chamber every day to minimize the effect of micro-environmental differences within the chamber. The single females were reared under these conditions until release of their second clutch. This one-generation acclimation period serves to minimize maternal effects. Two to four individuals were then isolated from the second clutch and the mother was removed from the beaker. The date of birth of the second clutch individuals was recorded, and then the individuals were reared until maturity (first deposition of eggs in the carapace) and the date recorded to allow an estimate of age at maturity.

Upon reaching maturity, individuals were placed under a Leica M1G5C microscope and the number of eggs in the carapace were counted to obtain an estimate of fecundity. Body size at maturity and behavioral data were determined using a Leica microscope and camera to obtain video recordings of mature individuals. Body size was measured from still frames isolated from the video. Behavior of individuals was assessed by placing them in a 16 mm diameter by 3 mm depth arena containing 700 μL of ADaM and recording movement within the arena for 20 sec. Individual trajectories were then tracked with ImageJ software (Schneider *et al.* 2012) using the MTrackJ plugin (Meijering 2008). Three aspects of behavior were quantified using the tracking data: 1) maximum velocity, 2) mean velocity, and 3) the standard deviation of velocity (which provides an estimate of the erratic nature of movement).

### Data Analysis

Estimates of the mutational bias (ΔM), a mutation parameter that describes the sign and magnitude of phenotypic change resulting from mutation, were obtained for each trait for each of the six genotypes by determining the mutation-line mean phenotype (z_M_) for each of the five MA lines, and the mean phenotype (z_0_) from control lines. The per-generation change in mean phenotype (R_m_) was estimated as the slope of the weighted least-squares regression of z_M_ on the line-specific number of generations of divergence where estimates of z_0_ were used to set the y-intercept and estimates of z_M_ were weighted by the inverse of their sampling variance. Estimates of ΔM were generated by scaling R_m_ by the control mean phenotype (z_0_). Because ΔM is a scaled metric, it facilitates comparisons between traits and genotypes that may have differed in initial mean phenotype.

In order to compare estimates of ΔM among traits, genotypes, and populations we used Kruskal-Wallis tests with our estimates of ΔM. Specifically, to assess variation in trait susceptibility to mutation we used estimates of the absolute value of ΔM, which represents the magnitude of phenotypic change in response to mutation. To assess variation in ΔM at the level of genotype and population we used the original estimates of ΔM, which represents both the magnitude and direction of phenotypic change in response to mutation.

Estimates of evolvability (CV_m_^2^), which describes the per-generation rate of input of new mutational variance, were generated by first scaling the raw data by the corresponding MA line mean. The scaled data for a genotype-specific trait was then subjected to variance partitioning using a random effects model under restricted maximum likelihood (REML) as implemented by the lme4 package (Bates *et al.* 2015) in Program R (R Core Team 2016). This procedure yielded estimates of the within- and among-line components of variance, which correspond to the environmental variance (V_e_) and genetic variance among MA lines (V_g_), respectively. Estimates of CV_m_^2^ for each genotype-specific trait were calculated by dividing V_g_ by the number of generations of mutational divergence averaged across all MA lines for a genotype. Because the data were scaled prior to analysis, the estimates of CV_m_^2^ we obtain are dimensionless and allow comparisons among traits and genotypes.

### Data Availability

Data are provided with this article as a supporting file (File S1). Supplemental material available at Figshare: https://doi.org/10.25387/g3.6799034.

## Results

### Mutational Bias and Evolvability

The majority of ΔM estimates for each genotype-specific trait were significant (Table 1; Table S2). The individual estimates varied by two orders of magnitude and varied in sign, with some traits increasing in response to mutation while other traits decreased. In contrast, the majority of CV_m_^2^ estimates measured after an average of 22 generations of divergence were not significantly different from zero (Table 1; Table S2). Significant estimates of CV_m_^2^ occurred solely within genotype GC. Specifically, age at maturity, egg number, max velocity, and mean velocity showed significant estimates of CV_m_^2^ within genotype GC (Table 1; Table S2).

**Table 1:** Trait specific estimates of Mutational bias (ΔM), Initial mean phenotype (mean of the control population) (z_o_), and Evolvability (CV_m_^2^). Abbreviations for population are: G = German population; I = Israel population. Abbreviations for genotypes are: A, B, C = Individual genotypes comprising each population. Abbreviations for traits are: AM = Age at maturity; Egg = Egg number at maturity; Size = Body length at maturity; MaxV = Maximum velocity; MeanV = Mean velocity; SDV = Standard deviation of velocity. GOD = Average number of generations of divergence

Population	Genotype	Trait	GOD	z_0_	ΔM	CV_m_^2^
G	A	AM	19.2	14.2	−0.0038	0.0000
G	A	Egg	19.2	6.2	−0.0037	0.0000
G	A	Size	19.2	3.1	−0.0001	0.0000
G	A	MaxV	19.2	49.8	−0.0438	0.0000
G	A	MeanV	19.2	10.3	−0.0400	0.0000
G	A	SDV	19.2	9.2	−0.0424	0.0000
G	B	AM	21.4	13.4	−0.0038	0.0000
G	B	Egg	21.4	4.9	0.0022	0.0000
G	B	Size	21.4	2.9	0.0005	0.0000
G	B	MaxV	21.4	25.4	−0.0275	0.0000
G	B	MeanV	21.4	4.7	−0.0257	0.0000
G	B	SDV	21.4	4.2	−0.0277	0.0000
G	C	AM	22.8	14.2	0.0030	0.0007
G	C	Egg	22.8	4.3	−0.0028	0.0053
G	C	Size	22.8	3.0	−0.0005	0.0000
G	C	MaxV	22.8	73.4	0.0006	4.9231
G	C	MeanV	22.8	16.7	0.0003	0.0048
G	C	SDV	22.8	13.7	−0.0015	0.0002
I	A	AM	24.8	14.8	−0.0013	0.0000
I	A	Egg	24.8	6.0	0.0017	0.0000
I	A	Size	24.8	2.9	0.0021	0.0000
I	A	MaxV	24.8	49.9	0.0085	0.0000
I	A	MeanV	24.8	7.8	0.0125	0.0000
I	A	SDV	24.8	9.2	0.0083	0.0000
I	B	AM	23.0	14.0	0.0007	0.0000
I	B	Egg	23.0	6.6	0.0033	0.0000
I	B	Size	23.0	2.9	0.0010	0.0000
I	B	MaxV	23.0	69.5	0.0048	0.0000
I	B	MeanV	23.0	14.9	0.0001	0.0000
I	B	SDV	23.0	14.3	0.0005	0.0000
I	C	AM	23.4	14.2	−0.0001	0.0000
I	C	Egg	23.4	6.5	0.0016	0.0000
I	C	Size	23.4	3.1	−0.0001	0.0000
I	C	MaxV	23.4	66.5	−0.0032	0.0000
I	C	MeanV	23.4	10.8	−0.0020	0.0000
I	C	SDV	23.4	11.7	−0.0003	0.0000

### Mutational Bias Among Traits

**Figure 1:**
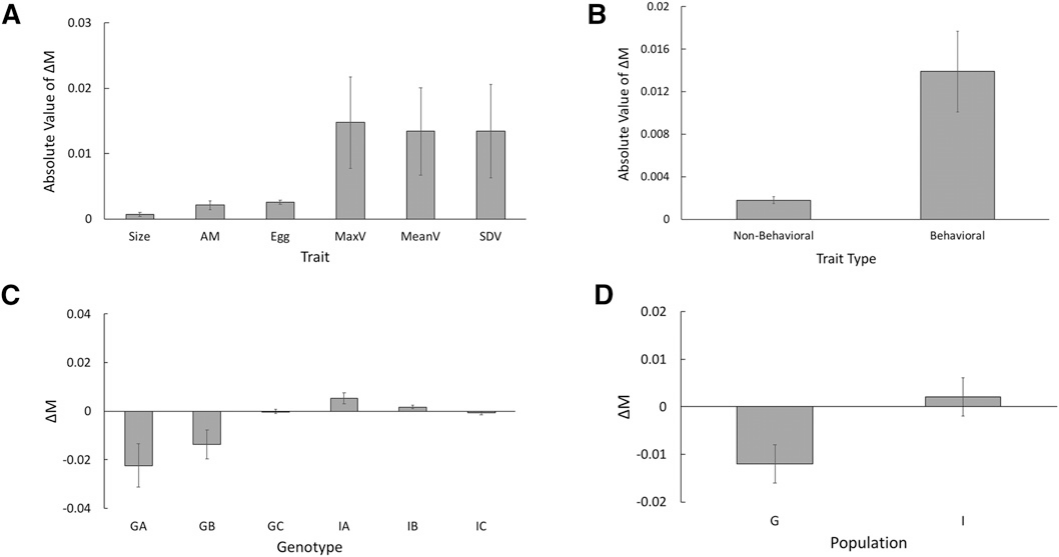
Mutational bias (ΔM) estimates among traits, trait type, genotypes, and populations. (A) Trait specific estimates of the absolute value of ΔM; (B) Trait type estimates of the absolute value of ΔM; (C) Genotypic estimates of ΔM; (D) Population estimates of ΔM. Error bars are ± SE. Size = Body length at maturity; AM = Age at maturity; Egg = Egg number at maturity; MaxV = Maximum velocity; MeanV = Mean velocity; SDV = Standard deviation of velocity. Non-behavioral = Size, AM, Egg; Behavioral = MaxV, MeanV, SDV. A, B, C = Individual genotypes comprising each population. G = German population; I = Israel population.

Estimates of the absolute value of ΔM, which represents the magnitude of phenotypic change, was high for maximum velocity, mean velocity, and standard deviation of velocity relative to age at maturity, body size, and egg number (Figure 1A). However, the estimates of ΔM did not significantly differ among individual traits (Figure 1A; Table 1; Table S3). When the individual traits were pooled into behavioral traits (max velocity, mean velocity, and standard deviation of velocity) and non-behavioral traits (age at maturity, body size, and egg number), behavioral traits displayed significantly larger estimates of the absolute value of ΔM than non-behavioral traits (Figure 1B; Table S3).

### Mutational Bias Among Genotypes

Estimates of ΔM varied significantly among individual genotypes comprising the German and Israel populations (Figure 1C; Table 1; Table S3). Overall, the three genotypes within the German population were characterized by negative estimates of ΔM for a majority of the traits examined (Table 1). Averaged across traits, two of the three genotypes (GA and GB) had overall negative estimates of ΔM (Figure 1C; Table 1; Table S3). In contrast, the trait-specific estimates of ΔM for the Israel genotypes were predominantly positive, resulting in genotypic estimates of ΔM that are positive for two of the three genotypes (IA and IB; Figure 1C; Table 1; Table S3).

### Mutational Bias Among Populations

Estimates of ΔM for the German and Israel populations, obtained by pooling the trait-specific estimates across all genotypes derived from each population, were significantly different (Figure 1C; Figure 1D; Table 1; Table S3). Specifically, the average estimate of ΔM for the German population was negative, indicating that trait values tend to decrease relative to the control following MA. Alternatively, the average estimate of ΔM for the Israel population was positive, suggesting trait values tend to increase relative to the control following MA.

## Discussion

Spontaneous mutations are the ultimate source of genetic variation, but our understanding of the context-dependence of mutation parameters is still limited. Environmental differences, genotypic variation and genetic architecture are all factors that influence estimates of spontaneous mutation rates and effects. For example, stressful environments can intensify or weaken mutational effects (Agrawal and Whitlock 2010). Additionally, traits that have complex genetic architectures are especially prone to spontaneous mutations because of their larger mutational target size (Houle 1992; Landry *et al.* 2007) Investigating both genotypic and trait-specific variability in mutational effects provides a means to understand the various context-dependencies of spontaneous mutations.

In the context of trait evolution by mutation, the magnitude of ΔM estimates is hypothesized to be directly proportional to the mutational target size for a trait. In our examination of trait evolution within *D. magna*, the absolute value of ΔM was higher for behavioral traits than other phenotypic traits. The greater sensitivity of behavioral traits (mean velocity, max velocity, and standard deviation of velocity) to spontaneous mutations suggests that the genetic architecture underlying these traits is more complex than the genetic architecture of life-history traits (age at maturity and egg number), and morphological traits (body size). Given that previous studies indicate life-history traits have a high susceptibility to mutation relative to morphological traits due to the complex genetic architecture associated with life-history traits (Houle *et al.* 1996; Latta *et al.* 2015), results from our phenotypic assay suggest behavioral traits may be more complex than life-history traits. These results agree with previous MA experiments that found behavioral traits are large mutational targets (Ajie *et al.* 2005; Estes *et al.* 2005). There was little evidence to suggest that significant mutational variability for the traits arose during the experiment, with only age at maturity, egg number, max velocity, and mean velocity in genotype GC producing significant estimates of CV_m_^2^ (Table S2). The limited divergence among MA lines, reflected by the large proportion of non-significant estimates of CV_m_^2^, is likely due to a combination of the low number of generations of divergence among MA lines for each genotype (Table 1), and the limited number of mutant lines associated with each genotype (five lines per genotype).

In general, variation in ΔM among genotypes indicates that genomic background may influence the rate at which phenotypic evolution occurs in the absence of selection. Specifically, the magnitude of ΔM may be an indicator of the underlying mutation rate (Remold and Lenski 2004; Weinreich *et al.* 2005; Phillips 2008; Le Gac and Doebeli 2010; Ness *et al.* 2015; Kronholm *et al.* 2017), while the sign of ΔM may indicate the context of the ancestral selective regimes. Estimates of ΔM also varied significantly among individual genotypes within *D. magna*. Two genotypes that originated from the German population (GA and GB) exhibited large negative estimates of ΔM, while genotype GC had a small negative estimate of ΔM. In contrast, genotypes IA and IB from the Israel population exhibited intermediate positive estimates of ΔM, while genotype IC was slightly negative. The variation in ΔM among genotypes observed here may reflect differences in the underlying mutation rate and/or type of selection acting on specific clonal genotypes. Genotypes that evolved quicker, genotypes GA, GB and IA, may have elevated mutation rates. These genotypes may have also experienced strong directional selection due to the local predation regime and may be optimally adapted to high predator densities. In contrast, genotypes that evolved slowly (GC, IB, and IC), may have lower underlying mutation rates, and also may be a result of stabilizing selection resulting from fluctuating selection that arises due to seasonal changes in predator density. Thus, these genotypes may be optimally adapted to intermediate or low predator densities. While these suppositions require further experimentation, they may help demonstrate the influence of fluctuating environments on genetic diversity within populations, and its influence on genotypic susceptibility to mutation.

A comparison of mutation parameter estimates from two ecologically divergent populations of *D. magna* indicated that the magnitude of ΔM in the German population was greater than the estimate from the Israel population, suggesting traits in the German population evolve under relaxed selection faster than traits in the Israel population. Additionally, the sign of ΔM for the German population is negative while the estimate from the Israel population is positive. Mutated lines among the Israel population displayed increased first egg number, increased body size, and higher velocities relative to non-mutated controls. A possible explanation for this seemingly adaptive trend requires an understanding of the ancestral environment of the *D. magna* used in the experiment. *Daphnia* that originate in environments containing visually-feeding predators experience selection that drives trait evolution to minimize detectability, resulting in small body size and small egg number (*e.g.*, Fisk *et al.* 2007), and minimal movement. When these *Daphnia* are released from selection, new mutations generate phenotypes that would be deleterious in the context of the ancestral environment, such as those observed in this study. Additionally, the increased velocities may have resulted from the release of selection from a warm thermal environment. Given that the Israel population originated from a warmer environment, individuals in this population likely maintain physiological function without the requirement of expending excess energy through behavior to generate metabolic heat. In the absence of selection, new mutations may result in unnecessary energy expended as movement. In contrast, the German population evolved smaller body size, smaller egg number and slower movement in the absence of selection. These results suggest the ancestral phenotypes in this population include large body size, large egg number, and faster movement. These ancestral phenotypes are characteristic of *Daphnia* populations that originate from an environment free of visual predators, but in which gape-limited ambush invertebrate predators predominate.

In summary, susceptibility to deleterious mutation varies at the trait, genotypic, and population levels. Variation at the level of traits is directly proportional to the putative mutational target size of the trait, with traits under the control of numerous genetic loci, such as behavior, displaying more susceptibility to mutation than traits under the control of few genetic loci. Additionally, genotypes and populations vary in their susceptibility to mutation, and this variation likely reflects a combination of variation in the underlying mutation rates and the unique selection histories among genotypes.

## References

[bib1] Agrawal A. F. Wang A. D., 2008 Increased transmission of mutations by low-condition females: evidence for condition-dependent DNA repair. PLoS Biol. 6: e30 10.1371/journal.pbio.0060030 18271627 PMC2235904

[bib2] Agrawal A. F. Whitlock M. C., 2010 Environmental duress and epistasis: how does stress affect the strength of selection on new mutations? Trends Ecol. Evol. 25: 450–458. 10.1016/j.tree.2010.05.003 20538366

[bib3] Ajie B. C. Estes S. Lynch M. Phillips P. C., 2005 Behavioral degradation under mutation accumulation in *Caenorhabditis elegans*. Genetics 170: 655–660. 10.1534/genetics.104.040014 15834141 PMC1450389

[bib4] Almbro M. Simmons L. W., 2014 Sexual selection can remove an experimentally induced mutation load. Evolution 68: 295–300. 10.1111/evo.12238 24372608

[bib5] Ávila V. Chavarrías D. Sánchez E. Manrique A. López-Fanjul C., 2006 Increase of the spontaneous mutation rate in a long-term experiment with *Drosophila melanogaster*. Genetics 173: 267–277. 10.1534/genetics.106.056200 16547099 PMC1461422

[bib6] Azevedo R. B. Keightley P. D. Laurén-Määttä C. Vassilieva L. L. Lynch M., 2002 Spontaneous mutational variation for body size in *Caenorhabditis elegans*. Genetics 162: 755–765. 12399386 10.1093/genetics/162.2.755 PMC1462287

[bib7] Baer C. F. Shaw F. Steding C. Baumgartner M. Hawkins A., 2005 Comparative evolutionary genetics of spontaneous mutations affecting fitness in rhabditid nematodes. Proc. Natl. Acad. Sci. USA 102: 5785–5790. 10.1073/pnas.0406056102 15809433 PMC556281

[bib8] Bataillon T., 2000 Estimation of spontaneous genome‐wide mutation rate parameters: whither beneficial mutations? Heredity 84: 497–501. 10.1046/j.1365-2540.2000.00727.x 10849074

[bib9] Bates D. Maechler M. Bolker B. Walker S., 2015 Fitting Linear Mixed-Effects Models Using lme4. J. Stat. Softw. 67: 1–48. 10.18637/jss.v067.i01

[bib10] Demerec M., 1937 Frequency of spontaneous mutations in certain stocks of *Drosophila melanogaster*. Genetics 22: 469. 17246857 10.1093/genetics/22.5.469 PMC1208982

[bib11] Denver D. R. Morris K. Lynch M. Thomas W. K., 2004 High mutation rate and predominance of insertions in the *Caenorhabditis elegans* nuclear genome. Nature 430: 679–682. 10.1038/nature02697 15295601

[bib12] Estes S. Ajie B. C. Lynch M. Phillips P. C., 2005 Spontaneous mutational correlations for life-history, morphological and behavioral characters in *Caenorhabditis elegans*. Genetics 170: 645–653. 10.1534/genetics.104.040022 15834140 PMC1450393

[bib13] Estes S. Phillips P. C. Denver D. R. Thomas W. K. Lynch M., 2004 Mutation accumulation in populations of varying size: the distribution of mutational effects for fitness correlates in *Caenorhabditis elegans*. Genetics 166: 1269–1279. 10.1534/genetics.166.3.1269 15082546 PMC1470770

[bib14] Eyre-Walker A. Keightley P. D., 1999 High genomic deleterious mutation rates in hominids. Nature 397: 344–347. 10.1038/16915 9950425

[bib15] Eyre-Walker A. Keightley P. D., 2007 The distribution of fitness effects of new mutations. Nat. Rev. Genet. 8: 610–618. 10.1038/nrg2146 17637733

[bib16] Fisk D. L. Latta L. C. Knapp R. A. Pfrender M. E., 2007 Rapid evolution in response to introduced predators I: rates and patterns of morphological and life-history trait divergence. BMC Evol. Biol. 7: 22 10.1186/1471-2148-7-22 17300733 PMC1805496

[bib17] Flynn J. M. Chain F. J. Schoen D. J. Cristescu M. E., 2016 Spontaneous mutation accumulation in Daphnia pulex in Selection-Free *vs.* Competitive Environments. Mol. Biol. Evol. 34: 160–173. 10.1093/molbev/msw234 27777284

[bib18] Haag-Liautard C. Dorris M. Maside X. Macaskill S. Halligan D. L., 2007 Direct estimation of per nucleotide and genomic deleterious mutation rates in Drosophila. Nature 445: 82–85. (erratum: Nature 453: 128) 10.1038/nature05388 17203060

[bib19] Halligan D. L. Keightley P. D., 2009 Spontaneous mutation accumulation studies in evolutionary genetics. Annu. Rev. Ecol. Evol. Syst. 40: 151–172. 10.1146/annurev.ecolsys.39.110707.173437

[bib20] Houle D., 1992 Comparing evolvability and variability of quantitative traits. Genetics 130: 195–204. 1732160 10.1093/genetics/130.1.195 PMC1204793

[bib21] Houle D. Morikawa B. Lynch M., 1996 Comparing mutational variabilities. Genetics 143: 1467–1483. 8807316 10.1093/genetics/143.3.1467 PMC1207413

[bib22] Huang W. Lyman R. F. Lyman R. A. Carbone M. A. Harbison S. T., 2016 Spontaneous mutations and the origin and maintenance of quantitative genetic variation. eLife 5: e14625 10.7554/eLife.14625 27213517 PMC4929002

[bib23] Keith N. Tucker A. E. Jackson C. E. Sung W. Lledó J. I. L., 2016 High mutational rates of large-scale duplication and deletion in *Daphnia pulex*. Genome Res. 26: 60–69. 10.1101/gr.191338.115 26518480 PMC4691751

[bib24] Kibota T. T. Lynch M., 1996 Estimate of the genomic mutation rate deleterious to overall fitness in *E. coli*. Nature 381: 694–696. 10.1038/381694a0 8649513

[bib25] Klüttgen B. Dülmer U. Engels M. Ratte H. T., 1994 ADaM, an artificial freshwater for the culture of zooplankton. Water Res. 28: 743–746. 10.1016/0043-1354(94)90157-0

[bib26] Kondrashov A. S. Houle D., 1994 Genotype-environment interactions and the estimation of the genomic mutation rate in Drosophila melanogaster. P. Roy. Soc. Lond. B. Bio. 258: 221–227. 10.1098/rspb.1994.0166 7886063

[bib27] Kraemer S. A. Böndel K. B. Ness R. W. Keightley P. D. Colegrave N., 2017 Fitness change in relation to mutation number in spontaneous mutation accumulation lines of *Chlamydomonas reinhardtii*. Evolution 71: 2918–2929. 10.1111/evo.13360 28884790 PMC5765464

[bib28] Kronholm I. Bassett A. Baulcombe D. Collins S., 2017 Epigenetic and Genetic Contributions to Adaptation in Chlamydomonas. Mol. Biol. Evol. 34: 2285–2306. 10.1093/molbev/msx166 28535256

[bib29] Lande R., 1994 Risk of population extinction from fixation of new deleterious mutations. Evolution 48: 1460–1469. 10.1111/j.1558-5646.1994.tb02188.x 28568413

[bib30] Landry C. R. Lemos B. Rifkin S. A. Dickinson W. J. Hartl D. L., 2007 Genetic properties influencing the evolvability of gene expression. Science 317: 118–121. 10.1126/science.1140247 17525304

[bib31] Latimer C. A. McGuigan K. Wilson R. S. Blows M. W. Chenoweth S. F., 2014 The contribution of spontaneous mutations to thermal sensitivity curve variation in *Drosophila serrata*. Evolution 68: 1824–1837. 10.1111/evo.12392 24576006

[bib32] Latta L. C. IV Peacock M. Civitello D. J. Dudycha J. L. Meik J. M., 2015 The phenotypic effects of spontaneous mutations in different environments. Am. Nat. 185: 243–252. 10.1086/679501 25616142

[bib33] Latta L. C. Morgan K. K. Weaver C. S. Allen D. Schaack S., 2013 Genomic background and generation time influence deleterious mutation rates in *Daphnia*. Genetics 193: 539–544. 10.1534/genetics.112.146571 23183667 PMC3567742

[bib34] Le Gac M. Doebeli M., 2010 Epistasis and frequency dependence influence the fitness of an adaptive mutation in a diversifying lineage. Mol. Ecol. 19: 2430–2438. 10.1111/j.1365-294X.2010.04664.x 20497320

[bib35] Lynch M. Blanchard J. Houle D. Kibota T. Schultz S., 1999 Perspective: spontaneous deleterious mutation. Evolution 53: 645–663. 10.1111/j.1558-5646.1999.tb05361.x 28565627

[bib36] Lynch M. Bürger R. Butcher D. Gabriel W., 1993 The mutational meltdown in asexual populations. J. Hered. 84: 339–344. 10.1093/oxfordjournals.jhered.a111354 8409355

[bib37] Lynch M. Conery J. Burger R., 1995 Mutation accumulation and the extinction of small populations. Am. Nat. 146: 489–518. 10.1086/285812

[bib38] Mallet M. A. Chippindale A. K., 2011 Inbreeding reveals stronger net selection on Drosophila melanogaster males: implications for mutation load and the fitness of sexual females. Heredity 106: 994–1002. 10.1038/hdy.2010.148 21119701 PMC3186252

[bib39] Mallet M. A. Kimber C. M. Chippindale A. K., 2012 Susceptibility of the male fitness phenotype to spontaneous mutation. Biol. Lett. 8: 426–429. 10.1098/rsbl.2011.0977 22090202 PMC3367732

[bib40] Meijering E., 2008 MTrackJ (ImageJ plugin). Biomedical Imaging Group Rotterdam, Erasmus MC—University Medical Center, Rotterdam, Netherlands http://www. imagescience. org/meijering/software/mtrackj.

[bib41] Miner B. E. De Meester L. Pfrender M. E. Lampert W. Hairston N. G., 2012 Linking genes to communities and ecosystems: *Daphnia* as an ecogenomic model. Proc. Biol. Sci. 279: 1873–1882. 10.1098/rspb.2011.2404 22298849 PMC3311900

[bib42] Ness R. W. Morgan A. D. Vasanthakrishnan R. B. Colegrave N. Keightley P. D., 2015 Extensive de novo mutation rate variation between individuals and across the genome of *Chlamydomonas reinhardtii*. Genome Res. 25: 1739–1749. 10.1101/gr.191494.115 26260971 PMC4617969

[bib43] Perfeito L. Fernandes L. Mota C. Gordo I., 2007 Adaptive mutations in bacteria: high rate and small effects. Science 317: 813–815. 10.1126/science.1142284 17690297

[bib44] Phillips P. C., 2008 Epistasis—the essential role of gene interactions in the structure and evolution of genetic systems. Nat. Rev. Genet. 9: 855–867. 10.1038/nrg2452 18852697 PMC2689140

[bib45] R Core Team, 2016 R: A language and environment for statistical computing. R Foundation for Statistical Computing, Vienna, Austria. URL https://www.R-project.org/.

[bib46] Remold S. K. Lenski R. E., 2004 Pervasive joint influence of epistasis and plasticity on mutational effects in *Escherichia coli*. Nat. Genet. 36: 423–426. 10.1038/ng1324 15072075

[bib47] Rifkin S. A. Houle D. Kim J. White K. P., 2005 A mutation accumulation assay reveals a broad capacity for rapid evolution of gene expression. Nature 438: 220 10.1038/nature04114 16281035

[bib48] Rowe L. Houle D., 1996 The lek paradox and the capture of genetic variance by condition dependent traits. P. Roy. Soc. Lond. B. Bio. 263: 1415–1421. 10.1098/rspb.1996.0207

[bib49] Schneider C. A. Rasband W. S. Eliceiri K. W., 2012 NIH Image to ImageJ: 25 years of image analysis. Nat. Methods 9: 671–675. 10.1038/nmeth.2089 22930834 PMC5554542

[bib50] Sharp N. P. Agrawal A. F., 2012 Evidence for elevated mutation rates in low-quality genotypes. P. Natl. Acad. Sci. 109: 6142–6146. 10.1073/pnas.1118918109 PMC3341077 22451943

[bib51] Sharp N. P. Agrawal A. F., 2013 Male‐biased fitness effects of spontaneous mutations in *Drosophila melanogaster*. Evolution 67: 1189–1195. 10.1111/j.1558-5646.2012.01834.x 23550766

[bib52] Weinreich D. M. Watson R. A. Chao L., 2005 Perspective: sign epistasis and genetic constraint on evolutionary trajectories. Evolution 59: 1165–1174. 16050094

[bib53] Zeyl C. Mizesko M. Visser J. A. G. M., 2001 Mutational meltdown in laboratory yeast populations. Evolution 55: 909–917. 10.1554/0014-3820(2001)055[0909:MMILYP]2.0.CO;2 11430651

